# Biological Characterization of 8-Cyclopropyl-2-(pyridin-3-yl)thiazolo[5,4-*f*]quinazolin-9(8*H*)-one, a Promising Inhibitor of DYRK1A

**DOI:** 10.3390/ph12040185

**Published:** 2019-12-17

**Authors:** Corinne Fruit, Florence Couly, Rahul Bhansali, Malini Rammohan, Mattias F. Lindberg, John D. Crispino, Laurent Meijer, Thierry Besson

**Affiliations:** 1 Normandie Univ, UNIROUEN, INSA Rouen, CNRS, COBRA UMR 6014, 76000 Rouen, France; corinne.fruit@univ-rouen.fr (C.F.); florence.couly@insa-rouen.fr (F.C.); 2 Department of Medicine, Division of Hematology/Oncology, Northwestern University, Chicago, IL 60611, USA; rbhansali91@gmail.com (R.B.); malini.rammohan@northwestern.edu (M.R.); j-crispino@northwestern.edu (J.D.C.); 3 College of Medicine, University of Illinois, Chicago, IL 60611, USA; 4 ManRos Therapeutics & Perha Pharmaceuticals, Perharidy Peninsula, 29680 Roscoff, France; lindberg@perha-pharma.com (M.F.L.); meijer@perha-pharma.com (L.M.); 5 Department of Biochemistry and Molecular Genetics, Northwestern University Feinberg School of Medicine, Chicago, IL 60611, USA

**Keywords:** thiazolo[5,4-*f*]quinazolin-9(8*H*)-one, CMGC kinases, DYRK family kinases, SH-SY5Y-Tau-4R cells, pre-B cells, quiescence

## Abstract

Dual-specificity tyrosine phosphorylation-regulated kinases (DYRKs) hyperactivity has been linked to the development of a number of human malignancies. DYRK1A is the most studied family member, and the discovery of novel specific inhibitors is attracting considerable interest. The 8-cyclopropyl-2(pyridin-3-yl)thiazolo[5,4-*f*]quinazolin-9(8*H*)-one (also called **FC162**) was found to be a promising inhibitor of DYRK1A and was characterized in biological experiments, by western transfer and flow cytometry on SH-SY5Y and pre-B cells. Here, the results obtained with **FC162** are compared to well-characterized known DYRK1A inhibitors (e.g., Leucettine **L41** and **EHT1610**).

## 1. Introduction

**Figure 1:**
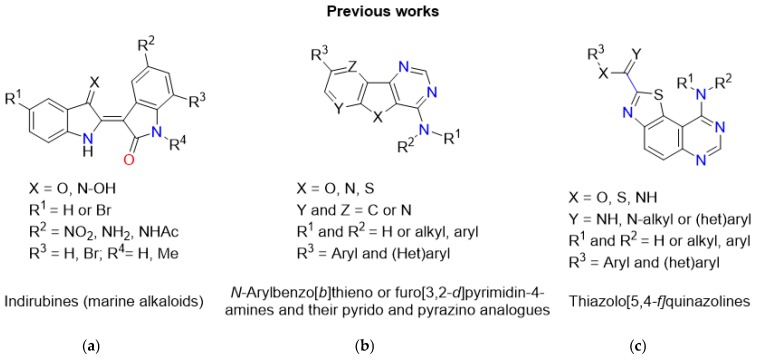
Summary of some of the compounds that have been identified by our group. (**a**) CDKs and GSK3 inhibitors [12,13]; (**b**) CK1 and CLK1 inhibitors [14,15,16]; (**c**) DYRKs inhibitors [17,18,19,20,21,22,23,24].

Protein phosphorylation catalyzed by kinases is a key cellular regulatory mechanism that is frequently dysregulated in human diseases [1]. This universal phenomenon controls major physiological events that are linked to the development of a variety of diseases such as diabetes [2,3], cancer [4,5], and neurodegenerative disorders [6,7,8]. Therefore, the search for new and efficient kinase inhibitors is a major aspect of drug discovery [9]. In the last 15 years, more than 40 kinases inhibitors have been approved by the US and Food and Drug Administration (FDA), mainly for cancer indications [10,11]. In the same period, our group has been dedicated to the conception and synthesis of bioactive heterocycles that can modulate the activity of deregulated kinases (Figure 1) [12,13,14,15,16,17,18,19,20,21,22,23,24,25,26,27], with a particular focus on dual-specificity tyrosine phosphorylation-regulated kinases (DYRKs) which play a role in the development of diseases such as cancer, Alzheimer’s disease (AD) and Down syndrome (DS) [28,29].

The DYRK family comprises DYRK1A/B, DYRK2, DYRK3, and DYRK4 and is itself included in the larger CMGC group that is composed of cyclin-dependent kinases (CDKs), mitogen-activated protein kinases (MAPKs), glycogen synthase kinases (GSKs) and CDC2-like kinases (CLKs) [30]. Due to its role in various diseases, DYRK1A is the most studied group member, and the discovery of specific inhibitors is attracting considerable interest [31,32].

In this context, our group focused its activity on the synthesis of novel angular thiazolo[5,4-*f*]quinazolin-9(8*H*)-one derivatives [25,26]. Innovative microwave-assisted metal-catalysed chemical reactions were studied [33,34], allowing the synthesis of important arrays of 2-aryl-*N8*-alkylthiazolo[5,4-*f*]quinazolin-9(8*H*)-ones which were screened on a panel of five kinases (CDK5/p25, CK1δ/ε (casein kinase 1), GSK-3α/β, CLK1 and DYRK1A, according to standard methods [35,36].

**Figure 2:**
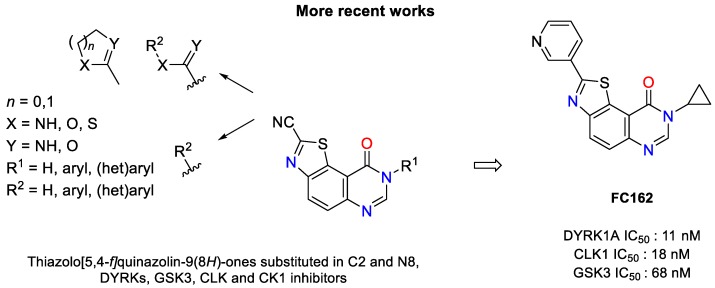
General aspect of the angular thiazolo[5,4-*f*]quinazolin-9(8*H*)-ones studied in our group, including the DYRK1A inhibitor **FC162** [25,26,27].

Among the various thiazolo[5,4-*f*]quinazolin-9(8*H*)-ones tested, only the 8-cyclopropyl-2-(pyridin-3-yl)thiazolo[5,4-*f*]quinazolin-9(8*H*)-one (also called **FC162**) (Figure 2) exhibited nanomolar IC_50_ values (11, 18, and 68 nM, against DYRK1A, CLK1, and GSK3, respectively) [27]. Compared to data obtained for other compounds, **FC162** was found to be the most promising candidate based on in vitro cell based assays.

Here we report that activity of **FC162** in two biological assays of DYRK1A function. These include testing the effect of **FC162** on Thr212-Tau phosphorylation and on growth of pre-B cells. Finally, the activity of **FC162** was compared to that of well-characterized chemicals known to be powerful DYRK1A inhibitors (e.g., Leucettine 41 (**L41**) [37,38] and **EHT1610** [22,39].

## 2. Results

### 2.1. Chemistry

The complete synthesis of **FC162** has been described in [27,34] (see SI for some Supplementary Materials). The target compound was obtained in eight steps from 5-nitroanthranilic acid in an overall yield of 14%, its percentage of purity was more than 99% (HPLC).

### 2.2. Biological Studies

#### 2.2.1. BBB Permeability Assay

As a complement to the preceding studies, the ability of **FC162** to cross the blood brain barrier (BBB) by passive diffusion was determined by PAMPA BBB assays with theophylline and corticosterone as standard compounds [40]. The results showed that the thiazolo[5,4-*f*]quinazolin-9(8*H*)-one **FC162** is able to cross the BBB by this transport process (Pe = 12.18 ± 1.10 × 10^−6^ cm.s^−1^), similarly to the lipophilic corticosterone (Pe = 13.86 ± 0.07 × 10^−6^ cm.s^−1^) (Table 1).

#### 2.2.2. Effect of FC162 on Thr212-Tau Phosphorylation in SH-SY5Y Cells

**Figure 3:**
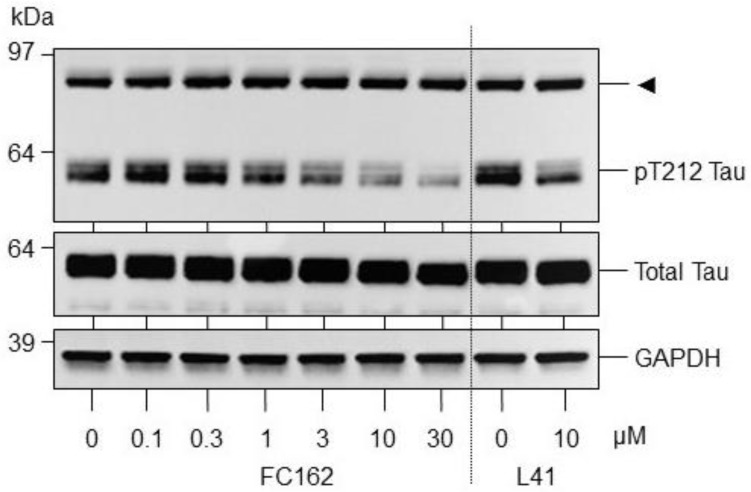
Inhibition of Tau Thr212 phosphorylation by **FC162.** SH-SY5Y cells expressing human Tau-4R were treated for 24 h with dimethylsulfoxide (DMSO) (0) or increasing concentrations of **FC162** or 10 μM of leucettine **L41** (used as a positive control). Isolated proteins were resolved by SDS-PAGE and analyzed by western blotting with antibodies directed against pT212-Tau, total Tau or glyceraldehyde-3-phosphate dehydrogenase (GAPDH) (used as loading control). The arrow indicates an unknown protein that cross-reacts with anti- pT212-Tau antibodies.

Thr212 phosphorylation is an excellent downstream measure of DYRK1A activity and/or inhibition. We have shown (unpublished) that in Tg (DYRK1A) mice (these animals express one extra copy of DYRK1A—the level of DYRK1A mRNA, protein, and kinase activity is multiplied by a factor of 1.5 [41]), the level of endogenous brain Tau phosphorylated on Thr212 is increased compared to that seen in corresponding wild-type mice. The SH-SY5Y neuroblastoma cell line overexpressing the four-repeat (4R) human Tau isoform (Tau-4R cells) was leveraged to analyze the effect of **FC162** on the phosphorylation of Thr212, a major DYRK1A phosphorylation site [42,43,44,45]. SH-SY5Y cells expressing Tau were exposed for 24 h to a range of **FC162** concentrations, harvested and their proteins resolved by SDS-PAGE, followed by western blotting with antibodies against p-Thr212-Tau, total Tau and GAPDH (loading control) (Figure 3). The well characterized DYRK1A inhibitor, Leucettine **L41** [37,38], was used as a reference compound. The results shown that there was a **FC162** dose-dependent inhibition of Tau phosphorylation at Thr212, further confirming the specific effect on DYRK1A in cells (Figure 3).

#### 2.2.3. DYRK1A-Specific Inhibitory Activity in Pre-B Cells

**Figure 4:**
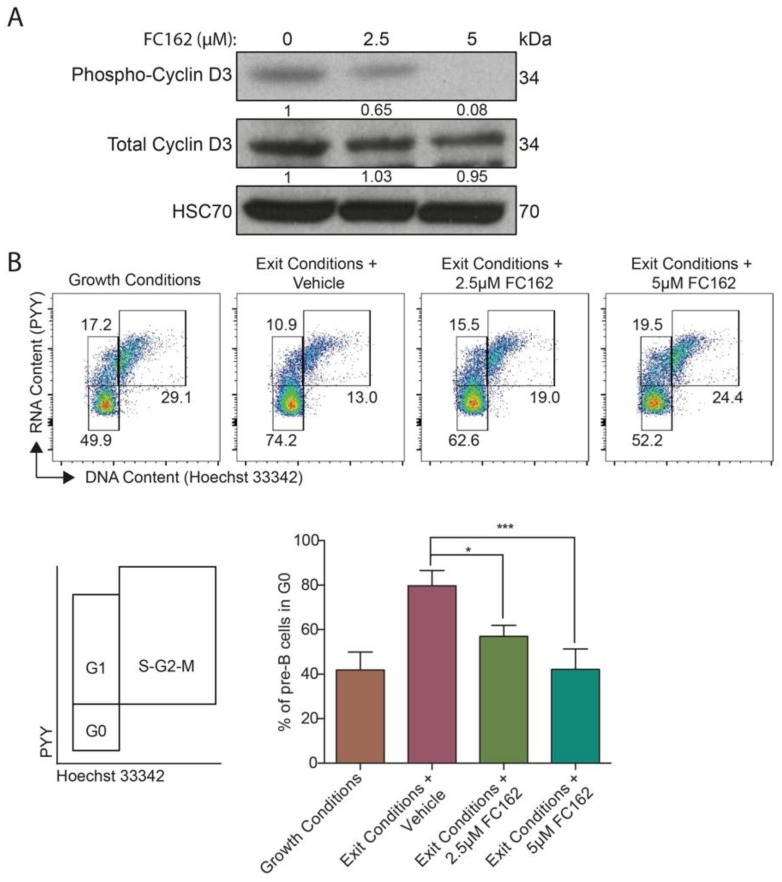
**FC162** reduces cyclin D3 phosphorylation and impairs the entry to quiescent state. (**A**) Western blot of protein extracted from murine pre-B cells cultured with vehicle or 2.5 and 5 μM **FC162** for 3 h. Densitometry values (below each lane) were normalized to HSC70. (**B**) Flow cytometry plots (upper), schematic (lower left), and bar graph (lower right) depicting the percentages of pre-B cells that have reached the quiescent state (defined as G0). Cells were grown either under growth conditions, which favor proliferation, or exit conditions, which promote quiescence. * *p* < 0.05, *** *p* < 0.001.

Using both a conditional DYRK1A knockout mouse and **EHT1610** a well described DYRK1A-specific inhibitor [20,21,22], we previously demonstrated that loss of DYRK1A activity decreases phosphorylation of cyclin D3 at Thr283 in pre-B cells and leads to stabilization of cyclin D3 and a subsequent drive out of the quiescent stage of the cell cycle [39]. To evaluate the cellular activity of **FC162** and its in vitro activity against DYRK1A, we assayed for changes in growth of primary mouse pre-B cells after treatment with the inhibitor (Figure 4).

Consistent with an on-target effect of **FC162**, we observed that the compound led to a reduction in cyclin D3 phosphorylation at Thr283 in a dose-dependent manner in murine pre-B cells (Figure 4A). Note that we did not see increased levels of cyclin D3 protein by 3 h, as previously shown [39]. However, when we assayed the cell cycle status of pre-B cells 48 h after the addition of **FC162** under conditions that favor cell cycle exit, we observed the expected decrease in the proportion of cells in G0 (Figure 4B). These results confirm that **FC162** treatment phenocopies the effect of *Dyrk1a* genetic deletion as well as **EHT1610** treatment.

## 3. Discussion

This work validates the utility of the thiazolo[5,4-*f*]quinazolin-9(8*H*)-one scaffold for the design of novel DYRK1A inhibitors. Through two biological assays, we found that **FC162** modified Tau phosphorylation and could alter cell cycle progression of pre-B cells. In both types of cellular in vitro studies, **FC162** was compared with two molecules (e.g., Leucettine **L41** and **EHT1610**) considered to be amongst the most potent DYRK1A inhibitors. In the context of these experiments the biological effect observed was found to be similar in both types, suggesting that **FC162** exerts its effects through DYRK1A inhibition. Then, we anticipate that this promising lead compound will aid in the design of more effective DYRK1A inhibitors.

## 4. Material and Methods

### 4.1. PAMPA-BBB Permeability Assay

These permeability assays were performed at the Centre d’Etudes et de Recherche sur le Médicament de Normandie (CERMN) in Caen, France. These assays were performed following the methodology developed by PION, by means of the Pampa-BBB Explorer™ system [40]. This system allows the measurement of the crossing velocity of a compound from one compartment to another through an artificial membrane at pH = 7.4. The experiment was replicated 6 times in 4 h, with quantification by UV-spectra reading. The result is given in Pe [cm.s^−1^]. The assayed compounds were diluted at 20 mM in DMSO, then diluted at 100 μM in Prisma HT Buffer pH 7.4 (pION). 200 μL of this solution was placed in the wells of the donor plate. 5 μM of BBB-1 Lipid was placed in the filters of the acceptor plate followed by 200 μL of Brain Sink Buffer to the wells of the acceptor plate. The sandwich was assembled and incubated for 4 h at room temperature without stirring, then separated, and the UV–vis spectra of the donor and acceptor compartments were determined using a plate reader (Tecan infinite M200, Männedorf, Switzerland). Pe were calculated with the PAMPA Explorer software v.3.7 (pION Inc., Billerica, MA, USA). Standard compounds used were corticosterone and theophylline.

### 4.2. Effect of FC162 Thr212-Tau Phosphorylation in SH-SY5Y Cells

#### 4.2.1. Culture and Treatment of Cell Lines

SH-SY5Y neuroblastoma cells overexpressing the four-repeat (4R) human tau isoform (gift from Dr. Fred Van Leuven) were cultured in Dulbecco’s modified Eagle medium (DMEM):Nutrient Mixture F-12 (DMEM/F-12, Gibco, c/o Invitrogen, Saint Aubin, France) containing 1% penicillin-streptomycin mixture (Gibco, c/o Invitrogen, Saint Aubin, France) and 10% fetal bovine serum (FBS, Gibco) in a humidified, 5% CO_2_ incubator at 37 °C. One day before treatment 1.10^6^ cells SH-SY5Y-Tau-4R cells were seeded into 60 mm dishes. **FC162** was then added at different concentrations, or Leucettine **L41** at 10 µM (with a final concentration of 0.1% DMSO) and cells were incubated for an additional 6 h before harvesting. Cells were scraped in cold PBS, centrifuged at 10,000× *g* for 5 min at 4 °C, and pellets were snap-frozen in liquid nitrogen and kept at −80 °C.

#### 4.2.2. Cell Lysis, Electrophoresis, and Western Blotting

Cell pellets were lysed in homogenization buffer and centrifuged (17,000× *g* for 10 min at 4 °C). Protein extracts were mixed (1:1 *v*/*v*) with sample buffer (2× NuPAGE LDS sample buffer, 200 mM DTT). Following heat denaturation, equal amounts of proteins (20 or 30 µg) were loaded on NuPAGE precast 4–12% Bis-Tris protein gels. Electrophoresis was run in MOPS buffer. Rapid blot transfers were performed at 2.5 A/25 V for 7 min. Membranes were blocked in a buffer containing milk (5% Regilait in Tris Buffered Saline with 0.1% Tween (TBST)) for 1 h. Membranes were then incubated with the antibodies against Tau (1/2000 in milk, overnight at 4 °C), phospho T212-Tau (1/2000 in milk, overnight at 4 °C) or GAPDH (2 h at RT, 1:30,000 dilution; Bio-Rad, Marnes-la-Coquette, France). Finally, membranes were incubated for 1 h, at RT with goat anti-rabbit or goat anti-mouse antibodies (Bio-Rad, Marnes-la-Coquette, France) and chemiluminescent detection was achieved with homemade ECL-Tris buffer (100 mM Tris pH 8.5, 0.009% H_2_O_2_, 0.225 mM p-coumaric acid, 1.25 mM luminol) with Fusion Fx7 camera software.

### 4.3. DYRK1A-Specific Inhibitory Activity in Pre-B Cells

CD19+ cells were isolated from murine total bone marrow using the EasySep positive-selection system (Stem Cell TechnologiesGrenoble, France). Cells were expanded in DMEM supplemented with 10% FBS (Hyclone, Illkirsh, France), 2 mM L-glutamine, 10 mM HEPES (pH 8), 1 mM sodium pyruvate, 55 µM ß-mercaptoethanol, 50 µg/mL gentamicin, and 1x Primocin (Invivogen, Toulouse, France) in the presence of 5 ng/mL murine IL-7 and 10 ng/mL murine SCF (PreproTech, Neuilly-Sur-Seine, France). Cells were replated every 2 days, maintaining a concentration of 2e6 cells/mL, and used for assays after 6 days of expansion.

#### 4.3.1. Immunoblotting

Pre-B cells were treated with **FC162** (doses indicated in Figure 4) or vehicle (0.1% DMSO) for 3 h. Cells were then collected and lysed for 30 min on ice in TENT buffer (50 mM Tris, pH 8, 2 mM EDTA, 150 mM NaCl, 1% Triton X-100) supplemented with 5 mM NaF, 2 mM NaVO_3_, 2 mM ß-glycerophosphate, 2 mM sodium pyrophosphate, and 1x complete protease inhibitor EDTA-free (Roche, Basel, Switzerland). Lysates were cleared by centrifugation for 10 min at 21,000× *g* at 4 °C. Protein lysates were denatured in LDS sample loading buffer (Life Technologies, Carlsbad, CA, USA) with 5% ß-mercaptoethanol at 95 °C for 5 min and electrophoresed on 4–12% Bis-Tris gradient gels (Life Technologies). Proteins were transferred to PVDF membranes and probed with primary antibodies for phospho-cyclin D3 Thr283 (ab55322, Abcam), total cyclin D3 (C-16, Santa Cruz Biotechnology, Inc, Dallas, TX, USA), and HSC-70 (B-6, Santa Cruz Biotechnology, Inc), and detected with HRP-conjugated secondary antibodies and ECL substrate (GE Healthcare, Marlborough, MA, USA). Immunoblots were performed in triplicate. Band densitometry values were calculated using ImageJ software.

#### 4.3.2. Cell Cycle Analysis

Wild-type pre-B cells were replated in complete culture media with 100-fold less IL-7 and SCF for 2 days in order to induce cell cycle exit. Cells were stained with 10 µg/mL Hoechst 33,342 (Life Technologies, Carlsbad, CA, USA) for 1 h in the dark at 37 °C prior to collection, washed, and resuspended in FACS buffer with 1 µg/mL Pyronin Y (Sigma Aldrich, St.-Louis, MO, USA) for 25 min before analysis. Cells were analyzed using LSRII flow cytometer (BD Bioscience-US, San Jose, CA, USA). Cell cycle analysis was performed in triplicate.

## Figures and Tables

**Table 1:** PAMPA-BBB permeability assay of **FC162** compared to theophylline and corticosterone

Product Name	Concentration (μM)	logPe	Pe (10^−6^ cm^−1^)	BBB Cross
**FC162**	100	−4.92 ± 0.04	12.18 ± 1.10	YES
Theophylline	250	−6.26 ± 0.03	0.55 ± 0.03	NO
Corticosterone	100	−486 ± 0.07	13.86 ± 2.20	YES

## Supplementary Materials

The following are available online at https://www.mdpi.com/1424-8247/12/4/185/s1.
