# Supplementary material for: Biological Characterization of 8-Cyclopropyl-2-(pyridin-3-yl)thiazolo[5,4-f]quinazolin-9(8H)-one, a Promising Inhibitor of DYRK1A
Source: Pharmaceuticals (Basel). 2019 Dec 17;12(4):185. doi: 10.3390/ph12040185 (PMC6958357; doi:10.3390/ph12040185)
Supplement: Supplementary file 1 [file pharmaceuticals-12-00185-s001.pdf]

Supporting Information

Biological Characterization of 8-Cyclopropyl-2-(pyridin-3-yl)thiazolo[5,4-*f*]quinazolin-9(8*H*)-one, a Promising Inhibitor of DYRK1A

Corinne Fruit[1], Florence Couly [1], Rahul Bhansali [2,3], Malini Rammohan [2], Mattias F. Lindberg [4], John D. Crispino[2,5], Laurent Meijer [4], and Thierry Besson*[,1]

1 Normandie Univ, UNIROUEN, INSA Rouen, CNRS, COBRA UMR 6014, 76000 Rouen, France ; corinne.fruit@univ-rouen.fr (C.F.); florence.couly@insa-rouen.fr (F.C.)

2 Department of Medicine, Division of Hematology/Oncology, Northwestern University (Chicago, IL); rbhansali91@gmail.com (R.B.); malini.rammohan@northwestern.edu (M.R.); j-crispino@northwestern.edu (J.D.C.)

3 College of Medicine, University of Illinois – Chicago (Chicago, IL)

4 ManRos Therapeutics & Perha Pharmaceuticals, Perharidy Peninsula, 29680-Roscoff, France; lindberg@perha-pharma.com (M.L.); meijer@perha-pharma.com (L.M.)

5 Department of Biochemistry and Molecular Genetics, Northwestern University Feinberg School of Medicine (Chicago, IL); j-crispino@northwestern.edu (J.D.C.)

* Correspondence: thierry.besson@univ-rouen.fr; +33 (0) 235-522-904 (T.B)

Table of contents

1. Chemistry work. S2

1.2. General methods S2

1.3. Last step of the synthesis of (FC162). S2

1.4. ^{1}H-NMR and ^{13}C-NMR of FC162 .... S3

1.5. Purity (HPLC) of FC162: Chromatogram and results. S4

1. Chemistry work

1.2. General methods

Materials were obtained commercially and used without further purification. All reactions were carried out under inert atmosphere of argon or nitrogen and monitored by thin-layer chromatography with silica gel 60 F254 precoated aluminum plates (0.25 mm). Visualization was performed with a UV light at 254 and 312 nm. Purifications were carried out by flash column chromatography system equipped with a dual UV − vis spectrophotometer (200 − 600 nm), a frion collector (176 tubes), a dual pistopump (1 − 200 mL/min, Pmax = 15 bar), allowing quaternary gradients and an additional inlet for air purge. Samples can be injected in liquid or solid mode. Melting points of solid compounds were measured on a Stuart melting point SMP3 with a precision of ±1.5 °C. IR spectra were recorded on a PerkinElmer Spectrum 100 series FT-IR spectrometer. Liquids and solids were applied on the section attenuated total reflectance (ATR) accessories. Absorption bands are given in cm − 1. ^{1}H, ^{13}C NMR spectra were recorded at 295 K on a Bruker AVANCE 300 MHz, at 300 MHz relative to $CDCl_3$ (residual solvent signals). Mass spectra analysis was performed by the Mass Spectrometry Laboratory of the University of Rouen. Mass spectra (ESI) were recorded with a Waters LCP 1er XR spectrometer. Microwave experiments were conducted at atmospheric pressure (RotoSYNTH, Milestone Srl, Italy) or in pressurized reactors (0 − 30 bar) (Monomode 300, Anton Paar, France) in commercial microwave reactors especially designed for synthetic chemistry. The purity of all tested compounds was determined by chromatographic analysis performed at 25 °C on Ultimate 3000 (Thermo Scientific, Les Ulis, France) with a quaternary pump equipped with a photodiode array detector (DAD) managed at 254 nm. Column was a Luna C18 (150 mm × 4.6 mm; 3 µm particle size) provided by Phenomenex (Le Pecq, France). The mobile phase was water (A) and acetonitrile (B) (v/v); starting condition is 90% A and 10% B, in which the solvent B changed to 10% to 90% in 4% by min. Flow rate was 0.5 mL/min, and 5 µL were injected. The percentage of purity of **FC162** was more than 98%.

1.3. Last step of the synthesis of (**FC162**)

Thiazolo[5,4-*f*]quinazolin-9(8*H*)-one **3** (0.341 mmol), copper iodide (0.065 g, 0.341 mmol, 1 equiv), and TBD (95 mg, 0.682 mmol, 2.0 equiv) in dry DMF (850 µL) were added to a 2 mL glass vial, which was sealed under argon atmosphere. The mixture was stirred under microwave irradiation at 120 °C for 10 min. Then, $Pd(OAc)_2$ (7.6 mg, 0.034 mmol, 10 mol%) and 3-iodopyridine (0.140 g, 0.682 mmol, 2.0 equiv) were added to the mixture and purged with argon. The reaction was then stirred under microwave irradiation at 120 °C for 5 h. The resulting solution was diluted with dichloromethane, and washed three times with a 5% aqueous ammonia solution, then with water and brine. The organic layer was dried over Na_2SO_4 and concentrated under vacuum. The crude product was purified by flash chromatography on silica gel with $MeOH/CH_2Cl_2$ as eluent (1/0 to 95:5; v/v), to afford the corresponding product.

*8-Cyclopropyl-2-(pyridin-3-yl)thiazolo[5,4-f]quinazolin-9(8H)-one (**FC162**).* Flash chromatography eluent ($MeOH/CH_2Cl_2$, 1/0 to 95:5; v/v), Beige powder; mp: 263-265 °C; IR (neat) ν_{max} 3059, 3012, 2114, 1659, 1588, 1449, 1344, 1295, 1024, 837 cm^{-1}; ^{1}H-NMR ($CDCl_3$, 25 °C, 300 MHz): δ_H 9.41 (d, J = 2.3 Hz, 1H, H_{Ar}), 8.75 (d, J = 4.9 Hz, 1H, H_{Ar}), 8.54-8.38 (m, 2H, H_{Ar} + H^4), 8.26 (s, 1H, H^7), 7.88 (d, J = 8.7 Hz, 1H, H^5), 7.48 (dd, J = 8.0 and 4.9 Hz, 1H, H_{Ar}), 3.45-3.26 (m, 1H, NCH), 1.36-1.24 (m, 2H, CH), 1.14-0.96 (m, 2H, CH). ^{13}C-NMR ($CDCl_3$, 75.4 MHz): δ_C 168.5 (C), 161.3 (C), 153.4 (C), 151.9 (CH), 148.7 (CH), 146.7 (CH), 146.6 (C), 134.7 (CH), 131.7 (C), 129.8 (C), 129.3 (CH), 126.6 (CH), 124.1 (CH), 116.4 (C), 29.8 (CH), 6.7(2 CH_2). HRMS (ESI$^+$): *m/z* calcd for $C_{17}H_{12}N_4OS$ $[M + H]^+$: 321.0807; Found: 321.0810.

1.4. ^{1}H-NMR and 13C-NMR of FC162

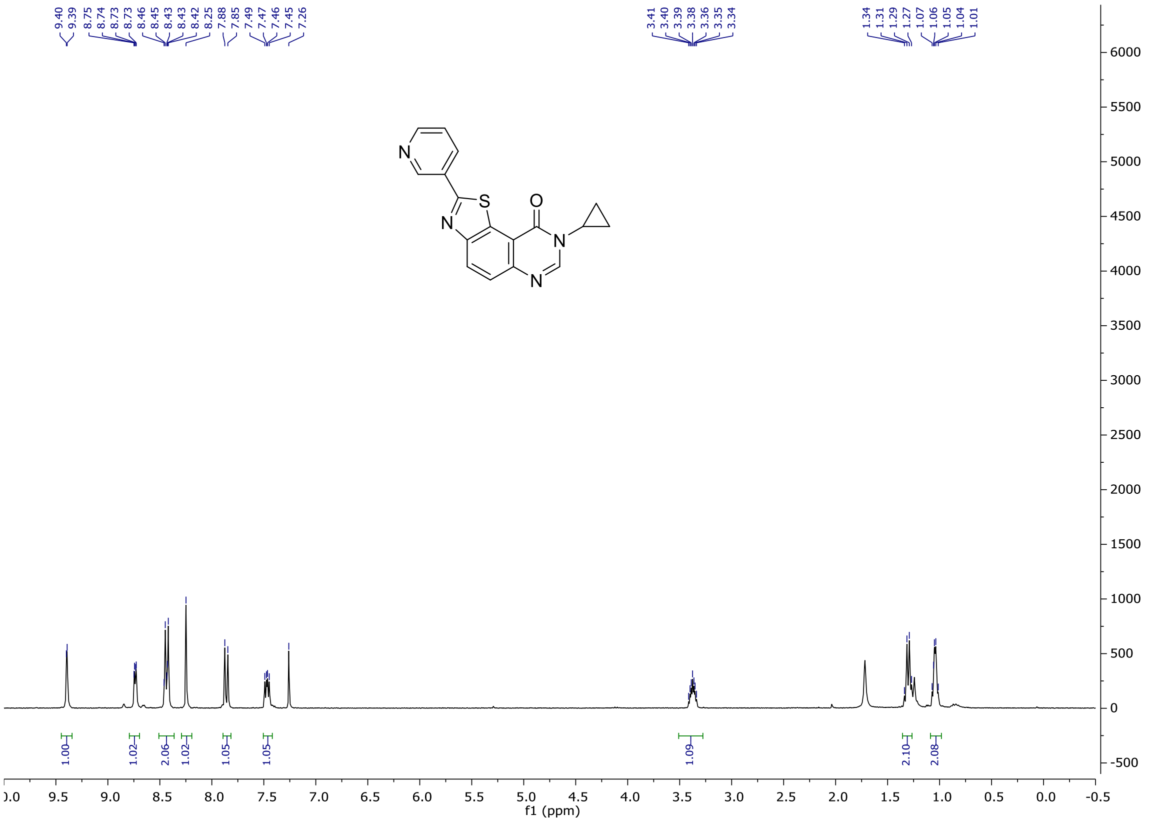

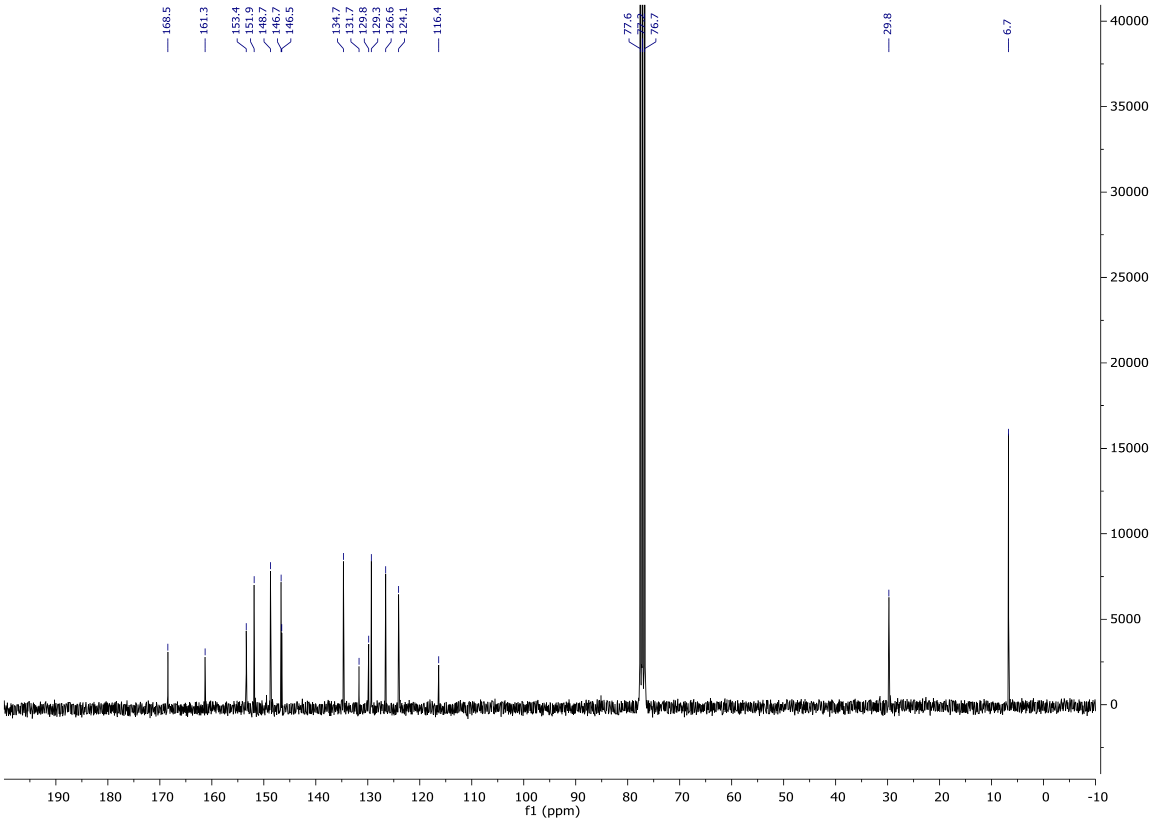

1.5. Purity (HPLC) of FC162: Chromatogram and results

Chromatogram and Results

Injection Details

Injection Name:	**FC162-1**	*Run Time (min):*	**45.00**
Vial Number:	**RA1**	*Injection Volume:*	**5.00**
Injection Type:	**Unknown**	*Channel:*	**UV_VIS_1**
Comment:	**Luna 3µm 11-07 rincage ACN**	*Wavelength:*	**254**
Instrument Method:	**grdt 10 a 90 en 20' - ACN-eau**	*Bandwidth:*	**2**
Processing Method:	**methode traitement**	*Dilution Factor:*	**1.0000**
Injection Date/Time:	**28/août/17 13:30**	*Sample Weight:*	**1.0000**

Chromatogram

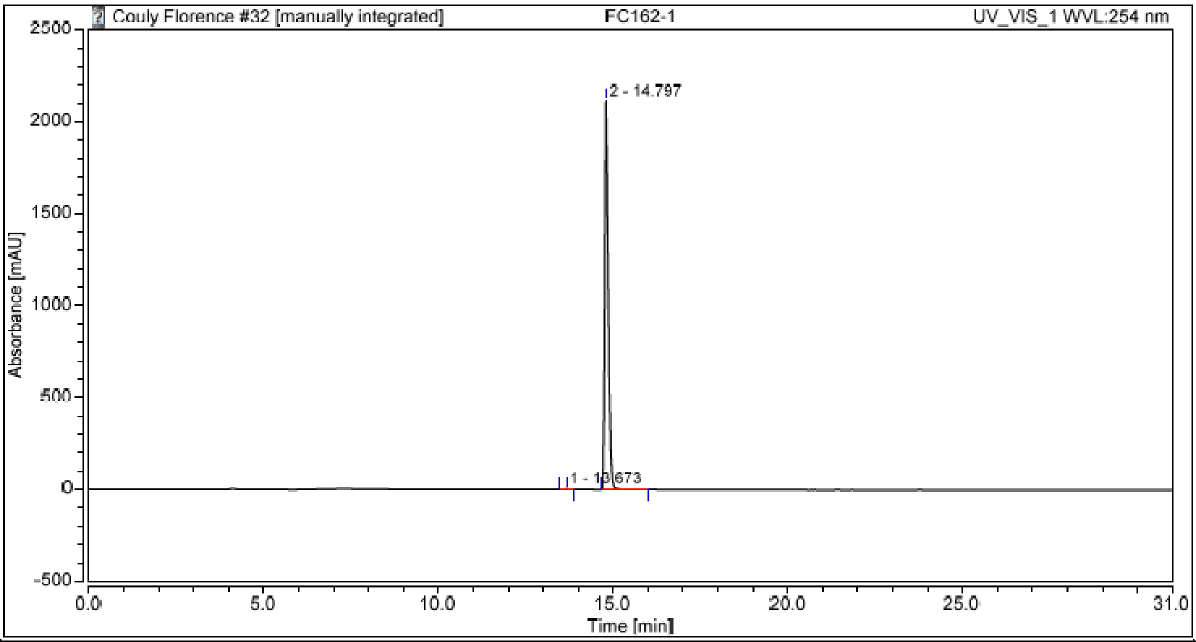

Integration Results

No.	Peak Name	Retention Time min	Area mAU*min	Height mAU	Relative Area %	Relative Height %	Amount n.a.
1		13.673	0.473	4.324	0.19	0.20	n.a.
2		14.797	243.566	2114.840	99.81	99.80	n.a.
**Total:**			**244.039**	**2119.164**	**100.00**	**100.00**	
